# Enhancing early Alzheimer's disease classification accuracy through the fusion of sMRI and rsMEG data: a deep learning approach

**DOI:** 10.3389/fnins.2024.1480871

**Published:** 2024-11-20

**Authors:** Yuchen Liu, Ling Wang, Xiaolin Ning, Yang Gao, Defeng Wang

**Affiliations:** ^1^School of Instrumentation Science and Opto-electronics Engineering, Beihang University, Beijing, China; ^2^Institute of Large-Scale Scientific Facility and Centre for Zero Magnetic Field Science, Beihang University, Beijing, China; ^3^Hefei National Laboratory, Hefei, China

**Keywords:** Alzheimer's disease, structural MRI, magnetoencephalography, deep learning, multimodal fusion

## Abstract

**Objective:**

Early detection and prediction of Alzheimer's Disease are paramount for elucidating neurodegenerative processes and enhancing cognitive resilience. Structural Magnetic Resonance Imaging (sMRI) provides insights into brain morphology, while resting-state Magnetoencephalography (rsMEG) elucidates functional aspects. However, inherent disparities between these multimodal neuroimaging modalities pose challenges to the effective integration of multimodal features.

**Approach:**

To address these challenges, we propose a deep learning-based multimodal classification framework for Alzheimer's disease, which harnesses the fusion of pivotal features from sMRI and rsMEG to augment classification precision. Utilizing the BioFIND dataset, classification trials were conducted on 163 Mild Cognitive Impairment cases and 144 cognitively Healthy Controls.

**Results:**

The study findings demonstrate that the InterFusion method, combining sMRI and rsMEG data, achieved a classification accuracy of 0.827. This accuracy significantly surpassed the accuracies obtained by rsMEG only at 0.710 and sMRI only at 0.749. Moreover, the evaluation of different fusion techniques revealed that InterFusion outperformed both EarlyFusion with an accuracy of 0.756 and LateFusion with an accuracy of 0.801. Additionally, the study delved deeper into the role of different frequency band features of rsMEG in fusion by analyzing six frequency bands, thus expanding the diagnostic scope.

**Discussion:**

These results highlight the value of integrating resting-state rsMEG and sMRI data in the early diagnosis of Alzheimer's disease, demonstrating significant potential in the field of neuroscience diagnostics.

## 1 Introduction

Alzheimer's disease (AD) constitutes a formidable healthcare challenge, marked by a progressive decline in cognitive function. This decline typically begins with memory impairment and subsequently extends to affect behavior, speech, and motor skills. As the most prevalent form of dementia, AD currently affects approximately 50 million individuals globally, a number projected to double every two decades, potentially reaching 152 million by 2050 (Dementia, 2024). Mild cognitive impairment (MCI) is recognized as the prodromal stage of AD, characterized by cognitive decline that does not yet significantly disrupt daily activities (Petersen Ronald, 2011). Although a cure for AD remains elusive, early detection is critical for optimizing management strategies and may delay the progression to dementia. The identification of accurate and sensitive biomarkers associated with brain alterations in dementia is essential for facilitating early-phase clinical trials.

Neuroimaging, particularly Structural Magnetic Resonance Imaging (sMRI), plays a crucial role in evaluating brain structure, specifically the volume of gray matter in regions impacted by AD, such as the medial temporal lobes (Frisoni et al., 2010). However, atrophy often signifies late-stage changes, manifesting years after the initial molecular alterations (Frisoni et al., 2017; Woo et al., 2017). Magnetoencephalography (MEG) presents a promising alternative for the identification of functional biomarkers in early-stage AD, owing to its superior temporal resolution and reliability, which are not confounded by neurovascular effects (Hornero et al., 2008; Schoonhoven et al., 2022). In contrast to Electroencephalography (EEG), MEG offers enhanced spatial resolution, facilitating the detection of alterations in the brain's functional connectome. This capability is vital for the application of machine learning techniques aimed at distinguishing features of MCI (Maestú et al., 2015).

Recent years have seen a proliferation of sophisticated computer-aided diagnosis techniques leveraging Artificial Intelligence (AI) for the accurate diagnosis and classification of Alzheimer's disease (AD) and other forms of dementia. Lopez-Martin et al. (2020) introduced a deep learning model utilizing synchrony measurements from MEG to detect early symptoms of Alzheimer's disease. This model, a novel deep learning architecture based on random block ensembles, processes neural activity-reflected magnetic signal characteristics through a series of 2D convolutions, batch normalization, and pooling layers. Zhu et al. (2021) proposed a deep learning network named DA-MIDL, which employs local brain atrophy areas to extract discriminative features, combined with multi-instance learning and a global attention mechanism, for the early diagnosis of Alzheimer's disease and mild cognitive impairment. Giovannetti et al. (2021) proposed a new Deep-MEG method, transforming MEG data into an image-based representation and employing an ensemble classifier based on deep convolutional neural networks for the prediction of early Alzheimer's disease (AD). Fouad and El-Zahraa M. Labib (2023) explored the use of EEG signals for the automatic detection of Alzheimer's disease, demonstrating the superior performance of deep learning over traditional machine learning methods such as Naive Bayes and Support Vector Machines. Nour et al. (2024) proposed a novel method combining Deep Ensemble Learning (DEL) and a two-dimensional Convolutional Neural Network (2D-CNN) for the accurate diagnosis and classification of Alzheimer's disease (AD) and healthy controls (HC) through EEG signals. The proposed method achieved superior performance compared to traditional machine learning methods, demonstrating the potential of deep learning in the early detection of Alzheimer's disease.

Multimodal classification methods that utilize different modalities offer significant advantages over traditional single-modality-based approaches for diagnosing AD and its prodromal stage, MCI. The integration of complementary information from various imaging modalities can enhance the comprehensive understanding of AD-associated changes and improve diagnostic accuracy. For instance, Ferri et al. (2021) detected Alzheimer's disease patients using artificial neural networks and stacked autoencoders from resting-state EEG (rsEEG) and structural MRI (sMRI) variables, achieving classification accuracies of 80% (EEG), 85% (sMRI), and 89% (both). Deatsch et al. (2022) developed a generic deep learning model to differentiate between Alzheimer's disease patients and normal controls through neuroimaging scans, evaluating the impact of imaging modalities and longitudinal data on performance. The study revealed that models trained on ^18^F-FDG PET outperformed those trained on sMRI, and incorporating longitudinal information into the ^18^F-FDG PET model significantly enhanced performance. Qiu et al. (2024) proposed a Multi-Fusion Joint Learning (MJL) module to enhance the model's discriminative capability in AD-related brain regions by integrating PET and sMRI features across multiple scales. Xu et al. (2023) introduced a multilevel fusion network for identifying MCI using multimodal neuroimaging, achieving superior performance over existing methods by extracting local and global representations and establishing long-range dependencies.

The review of existing research clearly indicates a predominant reliance on unimodal methodologies in the field to date, despite the acknowledgment of multimodal techniques in prior discussions. An analysis of the progression of multimodal strategies underscores significant unresolved challenges. These include a constrained participant pool and the complexities associated with high-dimensional feature spaces, where the prevalent diagnostic strategy involves the mere amalgamation of multimodal features. This limitation hampers the advancement of multimodal classification techniques. Additionally, the diagnosis of patients with cognitive impairments frequently necessitates consideration of both brain atrophy and functional cognitive alterations. However, certain multimodal methodologies exclusively focus on structural transformations, disregarding functional variations. This oversight neglects the synergistic potential of multimodal imaging data. Vaghari et al. (2022b) highlighted the significance of rsMEG data in detecting MCI at an early stage, demonstrating that rsMEG can augment sMRI-based classification for MCI, thereby playing a crucial role in the preliminary identification of Alzheimer's disease. On this basis, this study endeavors to introduce an innovative deep learning-driven multimodal feature selection approach that not only minimizes irrelevant and redundant features but also harmonizes the complementary aspects of multimodal data.

In this study, we introduce a complex diagnostic network that utilizes sMRI and rsMEG modalities. Using the BioFind dataset, we developed an innovative CNN-transformer framework that combines cross attention mechanisms for feature fusion, aiming to improve the diagnosis of attention deficit disorder and prediction of mild cognitive impairment (MCI) through multimodal brain imaging. Our method uniquely employs sMRI and rsMEG as multimodal images and proposes the Spatial-Channel Cross-Attention Fusion (SCCAF) module. This module includes Multi-Modal Patch Embedding (MMPE) block to enhance the feature representation of multimodal data, Spatial-wise Cross-modal Attention (SCA) block to capture global feature correlations across multimodal data efficiently via cross-modal attention, and Channel-wise Feature Aggregation (CFA) block to dynamically integrate and fuses cross-modal data based on channel correlations, enabling the framework to discern non-local dependencies and amalgamate complementary cross-modal information effectively. Its superiority is validated through comparisons with single-modal methods, decision fusion methods, and reference comparison studies, demonstrating significant advancements in MCI progression prediction. Additionally, we delve into the rsMEG features of six frequency bands (delta, theta, alpha, beta, low gamma, or high gamma), enriching the comprehensiveness of the diagnostic model.

The specific contributions of this paper can be summarized as follows:

- We proposed a multimodal approach integrating sMRI and rsMEG for the enhanced early diagnosis and prediction of Alzheimer's disease (AD). To the best of our knowledge, this is the first study to utilize deep learning techniques to combine sMRI and rsMEG for disease diagnosis.
- We introduced an advanced diagnostic network that employs a Spatial-Channel Cross-Attention Fusion module for effective feature fusion within multimodal data. This method significantly outperforms unimodal approaches and other fusion techniques in both AD diagnosis and the prediction of mild cognitive impairment (MCI) progression.
- We performed a thorough comparative analysis of Alzheimer's diagnosis using sMRI and rsMEG images. This analysis encompassed unimodal comparisons, three fusion strategy comparisons, and an in-depth exploration of rsMEG features across six frequency bands, thereby demonstrating the diagnostic model's effectiveness and comprehensiveness.

## 2 Method

In this section, we begin by introducing the source of the dataset and detailing the dataset preprocessing steps. Subsequently, we provide a description of the proposed multimodal classification framework, including its constituent components and loss function.

### 2.1 Materials and preprocessing

Table 1 summarizes the sample used in this study. The BioFIND dataset (Vaghari et al., 2022a) was utilized, consisting of individuals with MCI and Healthy Controls (HC) from two sites: the MRC Cognition and Brain Sciences Unit (CBU) in Cambridge, England, and the Center for Biomedical Technology (CTB) in Madrid, Spain. Controls at CBU were selected from the CamCAN cohort and underwent health status verification through screening processes (Shafto et al., 2014). At CTB, controls underwent a comprehensive neuropsychological evaluation and received sMRI scans. MCI diagnosis at CTB followed the criteria set by Frisoni et al. (2011), which involved clinical assessment and quantitative metrics. After excluding cases with missing sMRI data and dental sMRI artifacts, the final dataset included 163 HC and 144 MCI records.

**Table 1:** Summary of data characteristics.

**Characteristic**	**HC**	**MCI**	**${T/X}^{2}$ and *p* value**
Site (CBU/CTB)	91/75	68/90	$X^{2}$ = 4.04, *p* = 0.04
Sex (M/F)	82/84	80/78	$X^{2}$ = 0.01, *p* = 0.91
Age (years)	71.3 (7.0)	72.9 (6,7)	T = -2.04, *p* = 0.04
Education (years)	14.5 (4.4)	10.8 (5.3)	T = 6.7, *p* < 0.001
MMSE	28.8 (1.2)	26.1 (2.8)	T = 11.11, *p* < 0.001
Recording duration (seconds)	481.5(262)	180.0(305)	Z = 4.19, *p* < 0.001
Recording hour (24 h)	12.8 (2.4)	12.6 (2.1)	T = 1.13, *p* = 0.26
Number of bad epochs	4.1 (2.8)	4.7 (3.8)	T = -1.74, *p* = 0.08

For Site and Sex, the data are presented as counts; for Recording Duration, medians are displayed alongside interquartile ranges (in parentheses). Means, accompanied by standard deviations (in parentheses), are provided for all other variables. Abbreviations used include: CBU for Cognition & Brain Sciences Unit; CTB for Center for Biomedical Technology; MMSE for Mini-Mental State Examination; MCI for Mild Cognitive Impairment; M for Male; F for Female; and SD for standard deviation.

The rsMEG data was acquired in magnetically shielded rooms at both sites. Environmental noise was suppressed through signal space separation utilizing MaxFilter 2.2.12 as described by Taulu and Kajola (2005). Data importation was facilitated by the SPM12 toolbox (available at http://www.fil.ion.ucl.ac.uk/spm/; Penny et al., 2011). A minimum of 120 s of resting-state data was used for all subjects. Data processing involved down-sampling to 500 Hz and band-pass filtering from 0.5 to 98 Hz. Artifact detection and the marking of bad epochs were performed through OSL's automatic detection mechanism, as outlined in Vaghari et al. (2022a). The analysis focused on sensor-level features without reconstructing the sources of the rsMEG data, specifically utilizing data from magnetometers (MAGs) due to their sensitivity to deeper brain signals, which is crucial in the study of Alzheimer's disease given its significant impact on brain structures (Garcés et al., 2017). Despite MAGs' susceptibility to noise, their selection was justified by their relevance in examining alterations in deep brain regions associated with Alzheimer's disease. The rsMEG data was segmented into a size of 102 × 8,192.

The sMRI data was collected using T1-weighted sMRIs. The CBU participants were scanned using a Siemens 3T TIM TRIO or Prisma MRI scanner using a Magnetization Prepared RApid Gradient Echo (MPRAGE) sequence with the following parameters: Repetition Time (TR) = 2250 ms; Echo Time (TE) = 2.99 ms; Inversion Time (TI) = 900 ms; flip angle = 9 degrees; field of view (FOV) = 256 mm × 240 mm × 192 mm; voxel size = 1 mm isotropic; and GRAPPA acceleration factor = 2. In contrast, the CTB participants were scanned on a General Electric 1.5 Tesla MRI scanner using a high-resolution antenna and a homogenization PURE filter, specifically employing a Fast Spoiled Gradient Echo sequence with the following parameters: TR/TE/TI = 11.2/4.2/450 ms; flip angle = 12 degrees; slice thickness = 1 mm; matrix size = 256 × 256; and FOV = 25 cm. The sMRI data was processed exclusively through diffeomorphic registration using the DARTEL toolbox (Ashburner, 2007) to the MNI152 template. The sMRI data was resized to a resolution of 192 × 192 × 182 voxels to align with the resolution of the rsMEG features.

### 2.2 Fusion strategies

**Figure 1:**
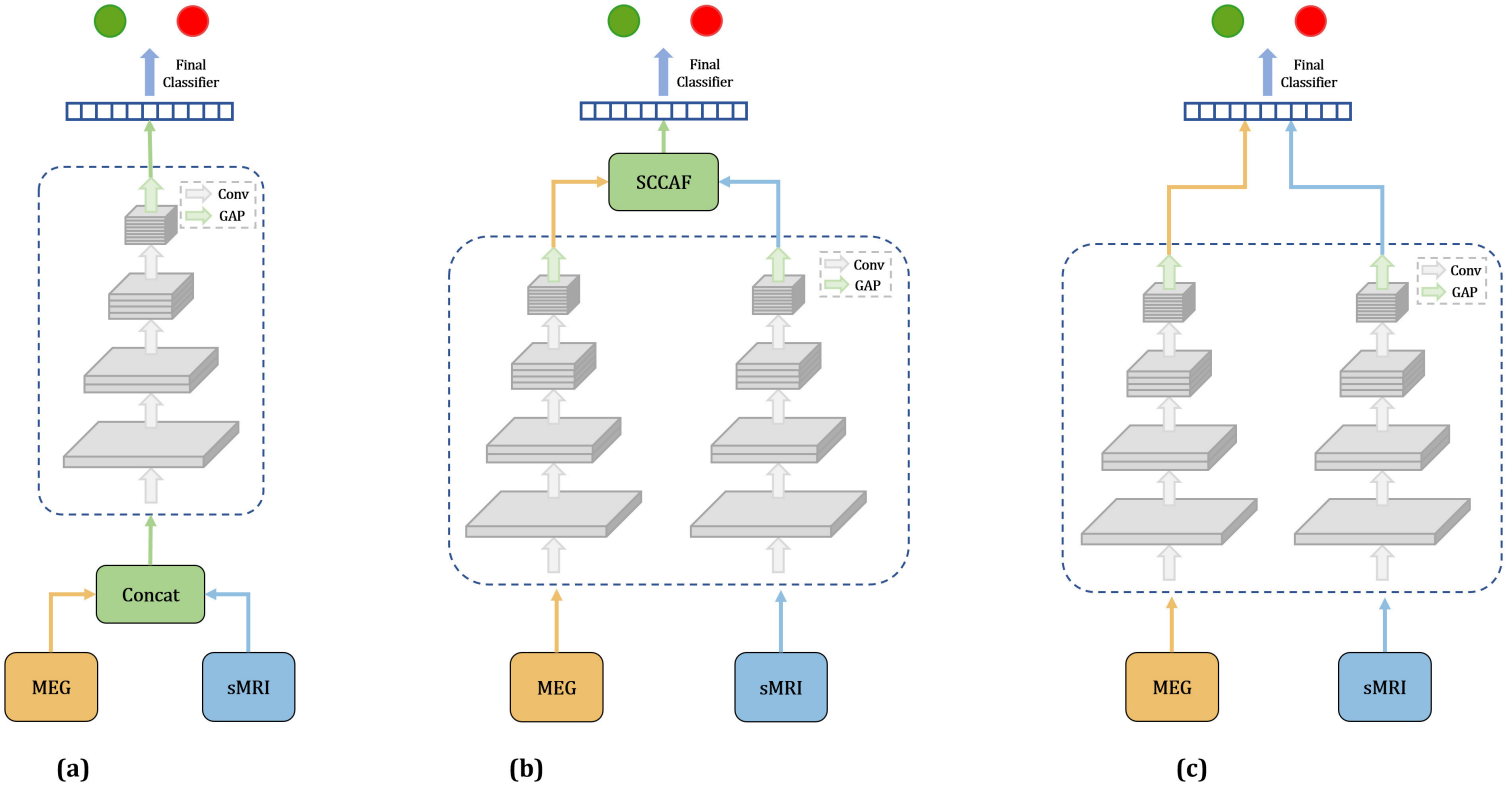
Overview of three fusion strategies. **(a)** Earlyfusion: Combines data from rsMEG and MRI before network processing. **(b)** Interfusion: Merges features extracted from these modalities using a specialized fusion module. **(c)** Latefusion: Combines the final outputs from each modality.

In this study, we investigate three distinct fusion strategies: EarlyFusion, InterFusion, and LateFusion, as illustrated in Figure 1, which provides a comprehensive overview of the methodologies employed.

**EarlyFusion:** EarlyFusion integrates raw data from various sources prior to feature extraction by the network. In this approach, MEG and sMRI data are downscaled to a uniform dimension and concatenated at the input layer. The fused information from these two modalities is subsequently processed by the feature extraction branch and classifier to generate the output. The feature extraction branches utilize four ResNet modules for feature extraction, comprising convolutional layers, normalization layers, and ReLU activation functions. The classifier produces the final output through two linear-ReLU layers.

**LateFusion:** LateFusion combines the individual classification results from each modality, which are then processed by a final classifier. Specifically, the rsMEG branch begins with a 1 × 15 strip convolution kernel for each channel, followed by a 3 × 3 kernel for inter-channel processing. The initial sMRI convolution employs a larger 7 × 7 × 7 kernel to achieve a wider receptive field and utilizes instance normalization to maintain inter-sample variance. Subsequent ResNet blocks for both branches incorporate downsampling layers to reduce feature size, with specific strides and kernel dimensions tailored to each modality, yielding rsMEG features of size (*C*, 102, 128) and sMRI features of size (*C*, 24, 24, 24), where *C* represents the number of channels. The features from both modalities are classified through two separate classifiers, with the final result produced by a concluding classifier. This approach allows for independent processing of features from each modality before their results are combined for classification.

**Figure 2:**
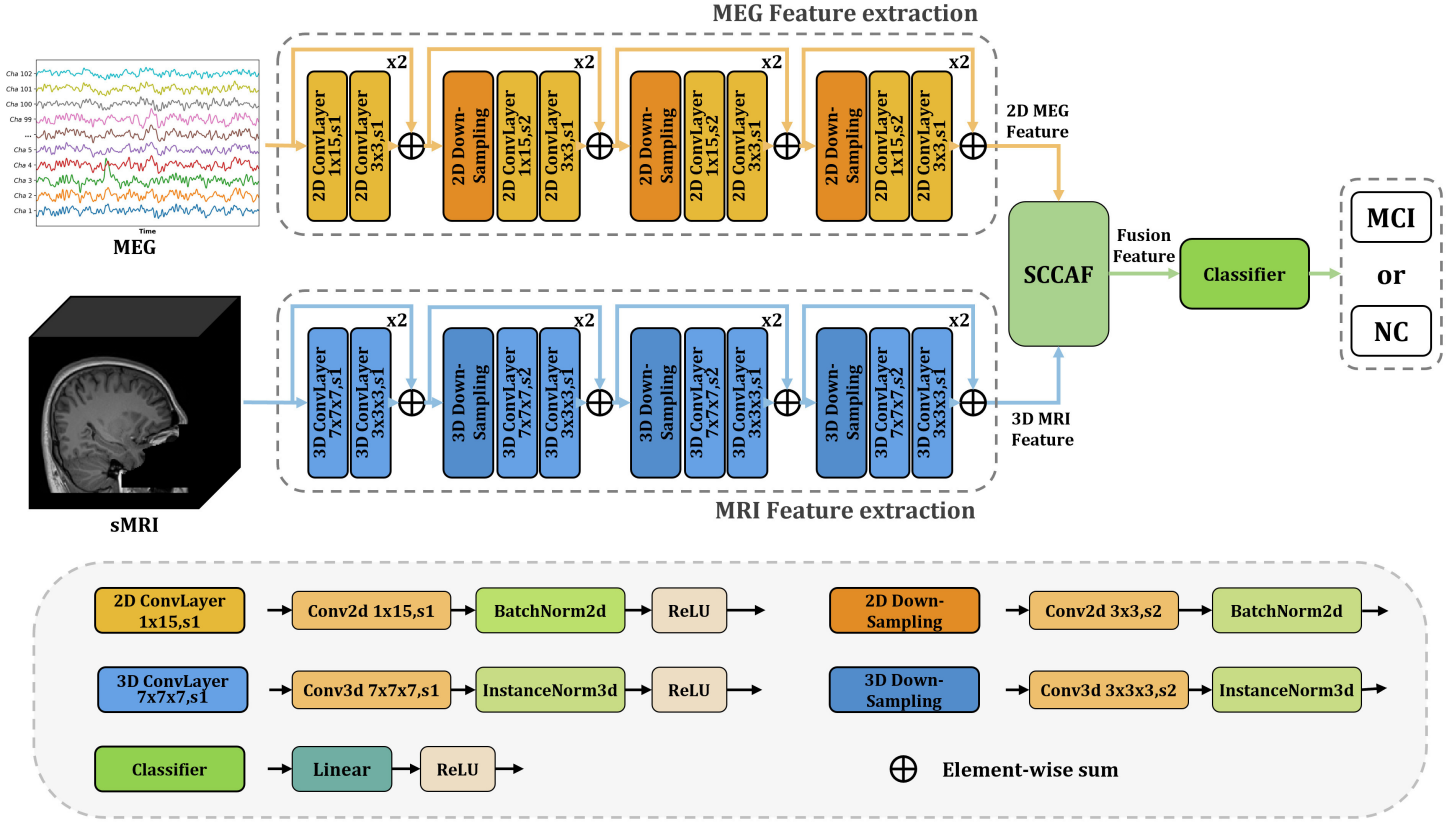
Overview of the proposed InterFusion multimodal classification framework for Alzheimer's disease. The proposed framework structure extends existing models from unimodal classification to multimodal scenarios. Our SCCAF module serves as a cross-modal solution to leverage multimodal complementarities.

**InterFusion:** InterFusion employs a dedicated fusion module to amalgamate the features extracted from these modalities. The proposed InterFusion network, as depicted in Figure 2, consists of four primary components: the rsMEG feature extraction branch, the sMRI feature extraction branch, the cross-attention fusion module, and the classifier. Unlike LateFusion, the extracted features are combined via a cross-attention fusion module, which captures the relationships between patterns, with the fusion results subsequently processed by a classifier. The specific structure of the fusion module will be detailed in the following subsection.

### 2.3 Spatial-channel cross-attention fusion module

**Figure 3:**
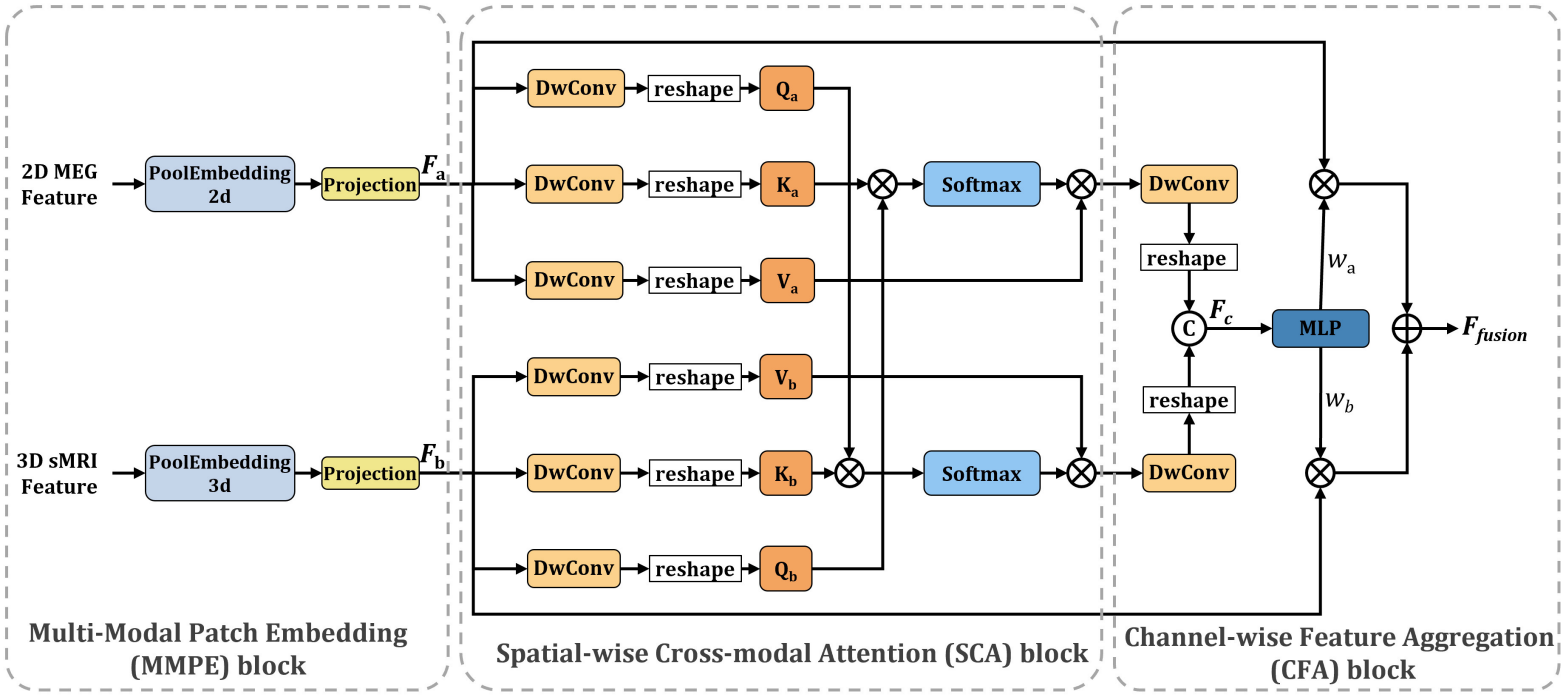
Overview of the proposed SCCAF module. Specifically, the SCCAF consists of MMPE to add position information and dimension expansion to the data, SCA to model global feature correlations among multimodal data and CFA to dynamically aggregate complementary features.

Figure 3 illustrates the Spatial-Channel Cross-Attention Fusion (SCCAF) Module. Initially, the module introduces a Multi-Modal Patch Embedding (MMPE) block designed to enhance the feature representation of multimodal data, thereby facilitating the subsequent cross-modal attention and feature aggregation processes. The procedure commences with patch extraction to merge rsMEG and sMRI features through the MMPE block. To reconcile feature dimension discrepancies, it applies GlobalAvgPool2d and GlobalAvgPool3d as initial steps, with pool sizes *P*_*s*1_ = (64, 64) for rsMEG features *F*_*rsMEG*_ and pool size *P*_*s*2_ = (16, 16, 16) for sMRI features *F*_*sMRI*_, respectively. Subsequently, it utilizes projection via kernel size 1 depth-wise convolutions on the flattened 2D and 3D patches, culminating in the concatenation of both feature sets:


(1) $$\begin{array}{l} \begin{array}{cc} F_{rsMEG} & =DwConv1d\left(flatten\left(GAP_{2d}\left(F_{rsMEG}\right)\right)\right),\\ F_{sMRI} & =DwConv1d\left(flatten\left(GAP_{3d}\left(F_{sMRI}\right)\right)\right), \end{array} \end{array}$$

where $F_{rsMEG},F_{sMRI}\in {\mathbb{R}}^{P\times C}$ represents the flattened fusion rsMEG and sMRI feature patches. *P* is calculated as 64 × 64 = 16 × 16 × 16 = 4096, which is the size of the flattened patches. *DwConv*1*d* represents depth-wise convolutions. *flatten* is used to reshape the input tensor into a 1-dimensional vector, and *GAP* symbolizes GlobalAvgPool, which represents the global average pooling operation.

To foster informative feature exchanges across modalities, the SCCAF module employs both Spatial-wise Cross-modal Attention (SCA) and Channel-wise Feature Aggregation (CFA) blocks. The SCA block leverages an enhanced cross-modal attention mechanism to map the global feature correlations between rsMEG and sMRI features. This block offers a broader receptive field compared to conventional CNN modules, thus aiding in the complementary aggregation of data. The advent of Transformer-based architectures (Dosovitskiy et al., 2020; Li et al., 2023) has demonstrated significant prowess in computer vision tasks, primarily through multi-head attention, which comprises several parallel non-local attention layers. The SCA Block processes a pair of images from different modalities, with its *Key* and *Value* derived from the same modality, while the *Query* originates from an alternate modality. For instance, the *Key* and *Value* might stem from the rsMEG modality, with the *Query* produced from the sMRI modality, and vice versa.


(2) $$\begin{array}{l} \begin{array}{cc} Z_{a} & =Softmax\left(\frac{Q_{b}K_{a}^{T}}{\sqrt{C}}\right)V_{a},\\ Z_{b} & =Softmax\left(\frac{Q_{a}K_{b}^{T}}{\sqrt{C}}\right)V_{b}, \end{array} \end{array}$$

where *a* indicates the rsMEG modality and *b* denotes the sMRI modality. The SCCAF block's output, derived by multiplying the *Value* by attention weights, elucidates the similarity between the *Query* in modality *b* and all *Key*s in another modality *a*, thereby aggregating and aligning information from both modalities.

To complementarily amalgamate cross-modal features based on their characterization capabilities, the CFA block is proposed to fuse cross-modal features and discern channel-wise interactions. Initially, it concatenates the SCA block outputs to obtain *F*_*c*_. Subsequently, it employs an MLP layer and softmax function to deduce the weight vectors $w_{a},w_{b}\in R^{C\times N}$, which recalibrate the rsMEG and sMRI features across channels. This process not only maximizes the utilization of aggregated information but also concurrently mitigates feature noise and redundancy. The final output of the CFA block is calculated as follows:


(3) $$\begin{array}{l} \begin{array}{cc} \left[w_{a},w_{b}\right] & =Softmax\left(MLP\left(F_{c}\right)\right),\\ F_{fusion} & =w_{a}\cdot F_{rsMEG}+w_{b}\cdot F_{sMRI}. \end{array} \end{array}$$

### 2.4 Loss function

In our study, we have chosen to use the CrossEntropyLoss to optimize our model. The CrossEntropyLoss calculates the loss by comparing the predicted probability distribution, denoted as *Q*, with the true probability distribution, denoted as *P*. It quantifies the information lost when using *Q* to approximate *P*. The formula for the CrossEntropyLoss is as follows:


(4) $$\begin{array}{l} L\left(P,Q\right)=\underset{x}{\sum }P\left(x\right)\log\left(\frac{1}{Q\left(x\right)}\right), \end{array}$$

where *P*(*x*) represents the true probability of class *x*, and *Q*(*x*) represents the predicted probability of class *x*. The loss is calculated for each class and summed up to obtain the total loss. By minimizing the CrossEntropyLoss, the model is guided to make more accurate predictions by reducing the divergence between the predicted distribution and the true distribution.

## 3 Experiments and results

In this section, we present the experimental setup and results of our multimodal classification framework. We begin by detailing the experimental setup, including the implementation details and the evaluation metrics. Subsequently, we provide a detailed analysis of the classification performance across unimodal models, different fusion strategies, rsMEG frequency bands in fusion and complexity analysis.

### 3.1 Implementation details

The proposed multimodal classification framework was implemented using the PyTorch deep learning library. Experiments were conducted at Dementias Platform UK. The network was trained using the Adam optimizer with a learning rate of 0.001, a batch size of 2, and a weight decay of 0.0001 over 100 epochs. Evaluation utilized a 5-fold cross-validation strategy, with the dataset partitioned into training and validation sets. Training data were utilized to train the network, while the validation set was used to evaluate its performance. Reported results represent the average of the five validation sets.

### 3.2 Evaluation metrics

In the evaluation of our model's performance, a comprehensive set of metrics was employed to ensure a holistic assessment. These metrics include Accuracy (ACC), F1-score, Sensitivity, Specificity, and the Matthews Correlation Coefficient (MCC).

- **Accuracy (ACC)** measures the proportion of true results (both true positives and true negatives) among the total number of cases examined. It is defined as:
(5) $$\begin{array}{l} ACC=\frac{TP+TN}{TP+TN+FP+FN}, \end{array}$$

where *TP*, *TN*, *FP*, and *FN* represent the numbers of true positives, true negatives, false positives, and false negatives, respectively.

- **F1-score**, is a measure that combines both precision and recall to provide a single value that conveys the balance between the two. It is given by:
(6) $$\begin{array}{l} \;Precision=\frac{TP}{TP+FP},\\ \;Recall=\frac{TP}{TP+FN},\\ \;F1\text{-}score=2\times \frac{Precision\times Recall}{Precision+Recall}, \end{array}$$
- **Sensitivity**, or recall, measures the proportion of actual positives correctly identified. The formula is:
(7) $$\begin{array}{l} Sensitivity=\frac{TP}{TP+FN}, \end{array}$$
- **Specificity** assesses the proportion of actual negatives that are correctly identified and is calculated as:
(8) $$\begin{array}{l} Specificity=\frac{TN}{TN+FP}, \end{array}$$
- The **Matthews Correlation Coefficient (MCC)** is a more informative measure of the quality of binary classifications, which takes into account true and false positives and negatives and is generally regarded as a balanced measure which can be used even if the classes are of very different sizes. The MCC is defined as:
(9) $$\begin{array}{l} MCC=\frac{TP\times TN-FP\times FN}{\sqrt{\left(TP+FP\right)\left(TP+FN\right)\left(TN+FP\right)\left(TN+FN\right)}}, \end{array}$$

### 3.3 Results

In this section, we present the results of our multimodal classification framework for Alzheimer's disease. Initially, we assessed the differences in classification performance between existing methods and our proposed method when using a single modality, either rsMEG or sMRI. Subsequently, we compared the impact of different data fusion strategies, including rsMEG only, sMRI only, and three different fusion strategies (EarlyFusion, InterFusion, and LateFusion), on classification performance. Furthermore, we conducted a comparison with Vaghari et al. (2022b)'s study to validate the effectiveness of our method in MCI prediction. Next, we investigated the performance of rsMEG features across six different frequency bands and the potential influence of these features when fused with sMRI data on classification performance. Lastly, we analyzed the complexity of the models.

#### 3.3.1 Evaluation of unimodal classification performance

We have developed unimodal networks specifically tailored for rsMEG and sMRI data to showcase the robust performance of our proposed method in single-modality settings. As shown in Table 2, our rsMEG-only network achieves superior performance metrics in comparison to other deep learning methods, attaining an accuracy of 0.710 and an F1-score of 0.615. Similarly, our sMRI-only network demonstrates exceptional performance, with an accuracy of 0.749 and an F1-score of 0.667. These metrics surpass those achieved by other methods. The notable improvements in F1-score and sensitivity underscore the efficacy of our method in accurately identifying relevant features. Our paired *t*-test results, adjusted using the Bonferroni correction, yielded significantly lower p-values (below 0.05), indicating that our method statistically significantly enhances performance.

**Table 2:** Comparison of the proposed method with other deep learning methods on unimodal data.

**Modal**	**Method**	**Accuracy**	**F1-score**	**Sensitivity**	**Specificity**	**MCC**	***P*-values**
rsMEG	EEGNet (Lawhern et al., 2018)	0.689	0.580	0.458	0.893	0.394	p ≤ 0.001
	WaveNet (Oord et al., 2016)	0.697	0.601	0.486	0.883	0.406	p ≤ 0.001
	2D ResNet-18 (He et al., 2016)	0.704	0.606	0.485	0.896	0.423	p ≤ 0.01
	2D EfficientNet-b0 (Tan and Le, 2019)	0.708	0.608	0.483	0.908	0.436	p ≤ 0.001
	Ours (rsMEG only)	0.710	0.615	0.493	0.902	0.437	-
sMRI	3D ResNet-18 (He et al., 2016)	0.726	0.625	0.486	0.939	0.483	p ≤ 0.01
	3D EfficientNet-b0 (Tan and Le, 2019)	0.731	0.637	0.503	0.933	0.489	p ≤ 0.01
	3D SENet (Hu et al., 2018)	0.730	0.639	0.510	0.923	0.482	p ≤ 0.001
	3D Unet (Ronneberger et al., 2015)	0.741	0.651	0.514	0.942	0.511	p ≤ 0.001
	Ours (sMRI only)	0.749	0.667	0.535	0.939	0.524	-

**Figure 4:**
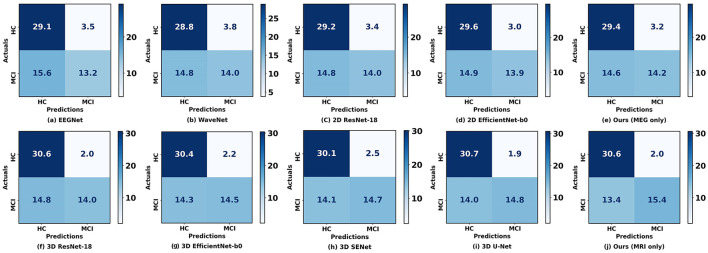
Confusion matrices between our proposed method and other methods when using a single modality. **(a–E)** Represent the confusion matrices using rsMEG data, while **(f–j)** represent the confusion matrices using sMRI data. The diagonal elements indicate the number of correctly classified samples, while the off-diagonal elements indicate the number of misclassified samples.

Figure 4 illustrates the confusion matrix comparison between our proposed method and other methods in classifying HC and MCI. Notably, our models utilizing rsMEG and sMRI data (e, j) exhibit superior performance. The rsMEG-only model demonstrates accurate identification of 29.4 HC and 14.2 MCI cases, while the sMRI-only model achieves even higher accuracy, correctly identifying 30.6 HC and 15.4 MCI cases. It is important to note that these results are obtained through 5-fold cross-validation, ensuring robustness and reliability in the evaluation process. These results underscore the robustness and precision of our unimodal networks in neuroimaging-based diagnostics.

#### 3.3.2 Effect of different fusion strategies on classification performance

Table 3 presents a comparative analysis of our proposed method against the reference method (Vaghari et al., 2022b), under various fusion strategies. Initially, our method demonstrates significant advantages compared to single-modality approaches. When using rsMEG and sMRI modalities independently, our method achieves accuracies of 0.710 and 0.749, respectively, and performs well across other metrics such as F1-score, sensitivity, specificity, and MCC. However, the performance is further enhanced with fusion strategies. Notably, under the InterFusion strategy, our method achieves the highest values in accuracy of 0.827, F1-score of 0.785, sensitivity of 0.678, specificity of 0.957, and MCC of 0.669 (*p* ≤ 0.001), indicating the efficacy of fusion strategies in improving model performance and validating the effectiveness of our proposed SCCAF Module. EarlyFusion achieves an accuracy of 0.756 (*p* ≤ 0.01), and LateFusion achieves 0.801 (*p* ≤ 0.05), both of which are improvements over using sMRI alone by 0.007 and 0.052, respectively, demonstrating the feasibility of multimodal fusion in providing more comprehensive information.

**Table 3:** Comparison of the proposed method with Vaghari et al.'s method on different fusion strategies.

**Method**	**Strategy**	**Accuracy**	**F1-score**	**Sensitivity**	**Specificity**	**MCC**	***P*-values**
Ours	rsMEG only	0.710	0.615	0.493	0.902	0.437	p ≤ 0.01
	sMRI only	0.749	0.667	0.535	0.939	0.524	p ≤ 0.001
	EarlyFusion	0.756	0.686	0.569	0.920	0.528	p ≤ 0.01
	InterFusion	0.827	0.785	0.678	0.957	0.669	p ≤ 0.001
	LateFusion	0.801	0.749	0.632	0.951	0.622	p ≤ 0.05
Vaghari et al.	rsMEG only	0.684	-	-	-	-	
	sMRI only	0.714	-	-	-	-	-
	EarlyFusion	0.698	-	-	-	-	-
	InterFusion	0.743	-	-	-	-	-
	LateFusion	0.772	-	-	-	-	-

Reference results directly sourced from Vaghari et al.'s study.

**Figure 5:**
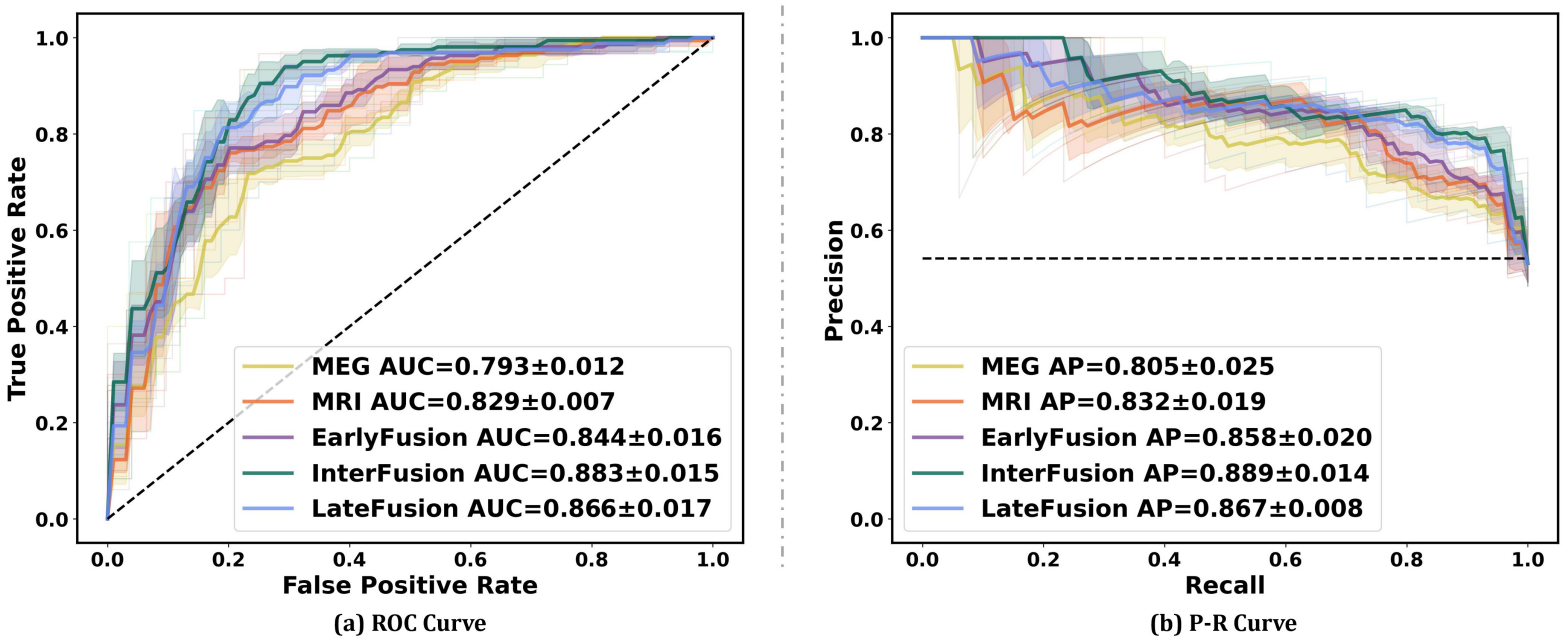
Performance on different data fusion strategies including rsMEG only, sMRI only, and three different fusion strategies (EarlyFusion, InterFusion, and LateFusion). Mean AUC was computed for each curve. The mean ROC/PR curve and its standard deviation are depicted as bold lines and shaded regions, respectively, in each plot. Dotted lines in each plot represent the classifier with random performance level.

Figure 5 illustrates the ROC curve and PR curve of different data fusion strategies, including rsMEG only, sMRI only, and three fusion strategies (EarlyFusion, InterFusion, and LateFusion). The results indicate that the InterFusion strategy outperformed both EarlyFusion and LateFusion, with an area under the receiver operating characteristic curve (AUC) of 0.883, surpassing EarlyFusion's AUC of 0.844 and LateFusion's AUC of 0.866. Similar trends are observed in the area under the precision-recall curve (AP), with InterFusion achieving 0.889. The consistency between AUC and AP performance underscores the robustness of our method on imbalanced datasets.

#### 3.3.3 Comparision with Vaghari et al.'s method

In this section, we present a comparative analysis between our proposed method and the method introduced by Vaghari et al. (2022b) using various fusion strategies. Table 3 provides a comprehensive comparison of the two methods in terms of accuracy, F1-score, sensitivity, specificity, and MCC. Regarding unimodality, Vaghari et al. achieves accuracies of 0.684 and 0.714 for the rsMEG and sMRI modalities, respectively. In contrast, our unimodality method significantly improves these metrics to 0.710 and 0.749, which is an improvement of 0.026 and 0.035, respectively. Furthermore, when considering fusion strategies, our method outperforms the reference results across all comparable strategies. Particularly, under InterFusion and EarlyFusion, our method achieves accuracies of 0.827 and 0.756, respectively, compared to Vaghari et al.'s 0.743 and 0.698. These improvements of 0.085 and 0.058, respectively, highlight the notable advancements of our approach in multimodal data fusion. Meanwhile, we note that our LateFusion and highest InterFusion are improved by 0.029 and 0.055, respectively, compared to the highest LateFusion of Vaghari et al. These results clearly demonstrate the superior efficacy of our method, proving the effectiveness of the deep learning method in multimodal fusion approaches.

#### 3.3.4 Analysis of the role of different frequency band features of rsMEG in InterFusion

In this investigation, we extend our analysis to encompass the exploration of rsMEG features across six distinct frequency bands: delta, theta, alpha, beta, low gamma, and high gamma. This detailed exploration aims to expand the depth and breadth of diagnostic models to provide a more nuanced and comprehensive understanding of MCI and AD diagnosis. As shown in Table 4, our research findings reveal interesting patterns and performance metrics for each frequency band. The High-Gamma frequency band (52–86 Hz) demonstrates the best results, with an accuracy of 0.818 and an F1-score of 0.775 (*p* ≤ 0.001). Its sensitivity is 0.670, and specificity is 0.948, indicating its potential in enhancing the diagnostic capabilities of the model, surpassing other frequency bands. The Delta frequency band (2–4 Hz) has an accuracy of 0.801 (*p* ≤ 0.01), which is the lowest among the six frequency bands. However, compared to the accuracy of 0.749 for MRI only, it shows an improvement of 0.052, indicating the effectiveness of combining rsMEG and MRI. Similarly, other encouraging results are observed, with an accuracy of 0.803 (*p* ≤ 0.001) for the Theta frequency band (4–8 Hz), 0.809 (*p* ≤ 0.01)for the Alpha frequency band (8–12 Hz), and 0.816 (*p* ≤ 0.05) for the Beta frequency band (12–30 Hz). The Low-Gamma frequency band (30–48 Hz) has an accuracy of 0.814 (*p* ≤ 0.01). It is worth noting that in terms of sensitivity, the high gamma frequency band (52–86 Hz) achieves the highest value of 0.677, demonstrating high gamma frequency band's potential in enhancing the diagnostic capabilities of the model.

**Table 4:** Exploring the rsMEG feature space in InterFusion.

**rsMEG frequency band**	**Accuracy**	**F1-score**	**Sensitivity**	**Specificity**	**MCC**	***P*-values**
Delta (2–4 Hz)	0.801	0.754	0.649	0.936	0.617	p ≤ 0.01
Theta (4–8 Hz)	0.803	0.758	0.656	0.933	0.619	p ≤ 0.001
Alpha (8–12 Hz)	0.809	0.764	0.656	0.945	0.634	p ≤ 0.01
Beta (12–30 Hz)	0.816	0.772	0.663	0.951	0.648	p ≤ 0.05
Low-Gamma (30–48 Hz)	0.814	0.774	0.677	0.936	0.640	p ≤ 0.01
High-Gamma (52–86 Hz)	0.818	0.775	0.670	0.948	0.650	p ≤ 0.001

#### 3.3.5 Complexity analysis

We also further analyzed the complexity of the model and compared the number of model parameters and floating point operations (FLOPs) between different fushion strategies, and the result is shown in Table 5. It is evident that although ResNet serves as the feature extraction backbone, the complexity of our work is significantly lower than that of 2D or 3D ResNet. This reduction in complexity is attributed to the smaller number of channels and layers in the feature extraction backbone. Specifically, InterFusion and LateFusion require the fusion of features extracted from two modalities, whereas EarlyFusion downsamples the two modalities before inputting them into the feature extraction branch. Consequently, the parameter count and FLOPs for InterFusion and LateFusion are higher than those for EarlyFusion, approximately equal to the sum of the parameters for rsMEG only and sMRI only. Additionally, it is noted that InterFusion has a little higher FLOPs than LateFusion due to the use of the SCCAF module. However, InterFusion has significantly much fewer parameters than LateFusion, indicating that the SCCAF module does not introduce excessive computational overhead, but rather reduces model parameters.

**Table 5:** Method complexity analysis.

**Method**	**Input size**	**#Param (M)**	**FLOPs (G)**
2D ResNet-18	(1,102,8192)	11.169	117.854
3D ResNet-18	(1,192,192,192)	33.141	707.935
rsMEG only	(1,102,8192)	3.811	17.164
sMRI only	(1,192,192,192)	7.105	108.145
EarlyFusion	(1,102,8192) & (1,192,192,192)	3.483	18.163
InterFusion	(1,102,8192) & (1,192,192,192)	9.235	138.251
LateFusion	(1,102,8192) & (1,192,192,192)	11.067	137.167

Greater values imply a greater degree of computational complexity.

## 4 Discussion

In this study, we explored the application of deep learning techniques in the early diagnosis of Alzheimer's disease, specifically through the fusion of rsMEG and sMRI data using a multimodal approach. Multimodal learning is a technique that combines multiple modalities through shared representations and has been successfully applied in various fields such as natural language processing, speech recognition, computer vision, and drug discovery. Recently, multimodal learning has been introduced in the field of medical imaging, with attention mechanisms and Transformer structures being applied in multimodal classification tasks. However, research on Transformers in medical tasks is still in its early stages, and previous studies have mostly used statistical or traditional methods to handle the discrimination task of MCI patients, highlighting the challenges posed by this problem compared to other classification tasks. In this work, we proposed a deep learning model that combines convolutional neural networks and cross-modal attention mechanisms to handle multimodal data and accurately identify Alzheimer's disease.

Firstly, we observed that the accuracy of using MRI data for classification is significantly higher than that of using rsMEG data. Based on Figure 4, compared to MRI data, the results using rsMEG data diagnosed more HC as MCI, with an average increase of 1.2 cases. This is not surprising as clinical doctors typically rely on MRI to support the diagnosis of MCI (Yang et al., 2021; Dubois et al., 2016; Frisoni et al., 2010). Although our rsMEG and MRI feature extraction branches are based on ResNet blocks, we achieved performance beyond ResNet by redesigning the convolutional kernel size and stride, highlighting the effectiveness of convolutional neural networks if devised properly.

Secondly, we compared different fusion strategies and found that the InteFusion method, which combines rsMEG and MRI data, achieved the best performance in multimodal classification, outperforming EarlyFusion or LateFusion. The main reason why the InterFusion method performs best in multi-modal classification tasks is primarily attributed to its ability to fully utilize the complementary information from both rsMEG and sMRI data. Specifically, rsMEG data provides supplementary information about functional activity and/or connectivity changes, while sMRI data provides structural information. Through our SCCAF module, the InterFusion method achieves effective cross-modal feature fusion and recognition of non-local dependencies in multi-modal feature representation. First, the InterFusion method employs the Multi-Modal Patch Embedding (MMPE) module, which performs initial pooling of rsMEG and sMRI features using GlobalAvgPool2d and GlobalAvgPool3d, and aligns the feature dimensions through deep convolutional projection. Second, the Spatial Cross-Modal Attention (SCA) module captures global feature correlations between rsMEG and sMRI features through an enhanced cross-modal attention mechanism, providing a wider receptive field that facilitates complementary data aggregation. Finally, the Channel Feature Aggregation (CFA) module dynamically fuses cross-modal features and adjusts feature weights through MLP layers and softmax function, maximizing the utilization of aggregated information while reducing feature noise and redundancy. Experimental results demonstrate that the InterFusion method outperforms other fusion strategies in terms of accuracy, F1-score, sensitivity, specificity, and MCC, validating the effectiveness of the SCCAF module in multi-modal fusion. Therefore, the InterFusion method significantly improves model performance by efficiently exchanging and fusing information between multi-modal data, demonstrating its superiority in addressing multi-modal medical classification problems.

Finally, our analysis of the rsMEG feature space in six frequency bands provides valuable insights into the performance of the diagnostic model. We found that low-frequency and high-frequency gamma waves performed the best, with accuracies of 0.814 and 0.818, respectively, demonstrating that rsMEG provides complementary information to MRI. This is consistent with previous M/EEG studies that emphasize the importance of gamma waves in research on MCI or genetic risk (Missonnier et al., 2010; Luppi et al., 2020). By integrating these different frequency bands, our method paves the way for a comprehensive understanding of MCI and AD, providing possibilities for improving diagnostic accuracy and clinical decision-making.

Recent multi-modal studies have highlighted the efficacy of integrating diverse neuroimaging techniques to enhance the classification and diagnosis of AD. sMRI offers intricate anatomical insights that facilitate the evaluation of brain structural alterations associated with neurodegeneration, particularly in the medial temporal lobes. In contrast, MEG captures the functional dynamics of brain activity in real-time, elucidating neural processes linked to cognition. The synergistic application of sMRI and MEG markedly enhances diagnostic precision for AD by amalgamating structural and functional data, fostering a more holistic understanding of the disease's trajectory. Compared to alternative multi-modal fusion strategies, such as the integration of sMRI with PET or EEG, the sMRI and MEG fusion paradigm is distinguished by MEG's superior temporal resolution and its ability to gauge brain activity independent of neurovascular coupling effects. This distinctive advantage positions the sMRI and MEG combination as a promising avenue for early diagnosis and monitoring of AD, potentially facilitating more effective intervention and management strategies.

In Vigari's studies, the authors primarily employed multi-kernel learning with support vector machines (SVM) for classification, utilizing LateFusion strategies akin to ensemble learning. While both studies examined three fusion methods, the features extracted and fusion techniques applied diverge significantly. Our approach capitalizes on deep learning methodologies, which inherently allow for more sophisticated feature extraction and representation learning. This foundational methodological divergence likely accounts for the discrepancies in our findings. Notably, our experimental results indicate that our fusion methods achieve significantly heightened accuracy compared to Vigari's work, underscoring the effectiveness of our approach and the advantages of deep learning in the context of disease diagnosis. This suggests that deep learning not only enhances the extraction of complementary information between sMRI and MEG but also augments classification performance in identifying Alzheimer's disease.

In our study, we employed the traditional convolutional network ResNet as the backbone for feature extraction from the two modalities, sMRI and MEG. This selection enables us to leverage ResNet's established efficacy in capturing spatial hierarchies and intricate patterns within imaging data. To optimize the information fusion process, we integrated the Spatial-Channel Cross-Attention Fusion Module, which adeptly amalgamates the complementary features from both modalities, yielding improved classification performance. While our current model exhibits promising results, there remains potential for further refinement. Future investigations could explore the integration of advanced transformer variants, such as convolutional adaptations of the vision transformer, which may confer advantages in multi-head learning and token-wise projections. This could facilitate more nuanced feature extraction and bolster model performance in differentiating among various stages of Alzheimer's disease.

Despite our notable accomplishments, we acknowledge certain limitations. Firstly, our current methodology does not account for information interaction during the feature extraction phase prior to fusion. Future research will investigate the incorporation of neighborhood and similarity information from the raw high-dimensional data across different imaging modalities. Secondly, we employed only one type of brain structural information; other imaging techniques, such as PET and fMRI, may yield superior results by directly measuring neurotransmitter levels or molecular pathology associated with attention deficit disorder, thereby further validating the significance of complementary information in multi-modal fusion. Future inquiries should also integrate non-imaging data to achieve a more comprehensive multi-modal approach. Qiu et al. (2022) utilized deep learning frameworks to process multi-modal data and execute multiple diagnostic steps, demonstrating diagnostic accuracy comparable to practicing neurologists and neuroradiologists. Recently, several classification frameworks have emerged that combine EEG and MRI fusion (Ferri et al., 2021; Colloby et al., 2016), with the future challenge lying in the integration of clinical insights with deep learning to elucidate changes in brain regions and establish a universal multi-modal classification model for disease diagnosis.

## 5 Conclusion

In this study, we propose a multimodal diagnostic network that utilizes sMRI and rsMEG modalities for the enhanced early diagnosis and prediction of AD and MCI. We introduce an innovative CNN-transformer framework that combines cross attention mechanisms for feature fusion, aiming to improve the accuracy of diagnosis and prediction through multimodal data. Our method uniquely employs sMRI and rsMEG as multimodal images and incorporates the SCCAF module, enabling effective fusion of complementary features and modeling of global feature correlations among multimodal data. Through extensive comparisons with single-modal methods, decision fusion methods, and different frequency band features of rsMEG in fusion, our results demonstrate the effectiveness of multimodal fusion in enhancing diagnostic accuracy and clinical decision-making, underscoring the potential of deep learning in multimodal medical imaging. Future research will focus on incorporating additional imaging modalities and non-imaging data to further enhance the diagnostic capabilities of our model and explore the potential of a unified multimodal model in clinical practice.

## Data Availability

The original contributions presented in the study are included in the article/supplementary material, further inquiries can be directed to the corresponding author.
